# Genomic analysis of natural intra-specific hybrids among Ethiopian isolates of *Leishmania donovani*

**DOI:** 10.1371/journal.pntd.0007143

**Published:** 2020-04-20

**Authors:** James A. Cotton, Caroline Durrant, Susanne U. Franssen, Tesfaye Gelanew, Asrat Hailu, David Mateus, Mandy J. Sanders, Matthew Berriman, Petr Volf, Michael A. Miles, Matthew Yeo

**Affiliations:** 1 Wellcome Sanger Institute, Hinxton, United Kingdom; 2 Faculty of Medicine, Addis Ababa University, Addis Ababa, Ethiopia; 3 Faculty of Infectious and Tropical Diseases, London School of Hygiene and Tropical Medicine, London, United Kingdom; 4 Department of Parasitology, Faculty of Science, Charles University, Prague, Czech Republic; Temple University, UNITED STATES

## Abstract

Parasites of the genus *Leishmania* (Kinetoplastida: Trypanosomatidae) cause widespread and devastating human diseases. Visceral leishmaniasis due to *Leishmania donovani* is endemic in Ethiopia where it has also been responsible for major epidemics. The presence of hybrid genotypes has been widely reported in surveys of natural populations, genetic variation reported in a number of *Leishmania* species, and the extant capacity for genetic exchange demonstrated in laboratory experiments. However, patterns of recombination and the evolutionary history of admixture that produced these hybrid populations remain unclear. Here, we use whole-genome sequence data to investigate Ethiopian *L*. *donovani* isolates previously characterized as hybrids by microsatellite and multi-locus sequencing. To date there is only one previous study on a natural population of *Leishmania* hybrids based on whole-genome sequences. We propose that these hybrids originate from recombination between two different lineages of Ethiopian *L*. *donovani* occurring in the same region. Patterns of inheritance are more complex than previously reported with multiple, apparently independent, origins from similar parents that include backcrossing with parental types. Analysis indicates that hybrids are representative of at least three different histories. Furthermore, isolates were highly polysomic at the level of chromosomes with differences between parasites recovered from a recrudescent infection from a previously treated individual. The results demonstrate that recombination is a significant feature of natural populations and contributes to the growing body of data that shows how recombination, and gene flow, shape natural populations of *Leishmania*.

## Author summary

Leishmaniasis is a spectrum of diseases caused by the protozoan parasite *Leishmania*. It is transmitted by sand fly insect vectors and is responsible for an enormous burden of human suffering. In this manuscript we examine *Leishmania* isolates from Ethiopia that cause the most serious form of the disease, namely visceral leishmaniasis, which is usually fatal without treatment. Historically the general view was that such parasites reproduce clonally, so that their progeny are genetically identical to the founding cells. This view has changed over time and it is increasingly clear that recombination between genetically different *Leishmania* parasites occurs. The implication is that new biological traits such as virulence, resistance to drug treatments or the ability to infect new species of sand fly could emerge. The frequency and underlying mechanism of such recombination in natural isolates is poorly understood. Here we perform a detailed whole genome analysis on a cohort of hybrid isolates from Ethiopia together with their potential parents to assess the genetic nature of hybrids in more detail. Results reveal a complex pattern of mating and inbreeding indicative of multiple mating events that has likely shaped the epidemiology of the disease agent. We also show that some hybrids have very different relative amounts of DNA (polysomy) the implications of which are discussed. Together the results contribute to a fuller understanding of the nature of genetic recombination in natural populations of *Leishmania*.

## Introduction

*Leishmania* is a diverse genus of kinetoplastid protozoan parasites from the family Trypanosomatidae. These parasites are best known as the cause of human and animal leishmaniasis, which is a clinically important neglected tropical disease affecting millions of people and causing a tremendous burden of mortality and morbidity [1,2]. Leishmaniasis comprises a spectrum of related diseases which, depending on the species, result in various presentations ranging from small, self-healing cutaneous lesions to widespread disseminated lesions, destructive mucosal and mucocutaneous pathology, and visceral disease that is usually fatal in the absence of effective chemotherapy [3]. *Leishmania* have a digenetic (two host) life cycle involving a vertebrate host and 166 different species of phlebotomine sand fly that have been implicated as vectors [4], although alternative invertebrate vectors may exist for some species [5]. Vertebrate hosts encompass a wide range of mammals or reptiles, and around 20 species of *Leishmania* have been reported to infect humans [4].

Historically, the population structure of *Leishmania*, other trypanosomatids and indeed most protozoan parasites was considered to be largely clonal [6]: the presumption was that admixture between members of the same clone, or between very closely related parasites was absent or rare, with minimal impact on population structure. However, at the time the clonal theory was first proposed, most population genetic data for trypanosomatids were based on inadequate sampling and use of low-resolution markers unlikely to detect admixture between genetic groups [7]. Subsequently, extensive work using multilocus sequence typing and microsatellite markers has produced a foundation for understanding the population genetics of some *Leishmania* species [7–10]. Most natural *Leishmania* isolates have surprisingly little heterozygosity, which has been widely ascribed to extensive selfing [11–13], although aneuploidy variation could also contribute [14]. In contrast there have also been a number of reports of heterozygous natural isolates possessing a mixture of alleles associated with different populations [15–17] and even different species [18,19], suggesting a hybrid origin of these isolates. There is therefore a growing body of evidence for genetic exchange in natural populations of several *Leishmania* species.

Laboratory genetic crosses between at least two *Leishmania* species have been achieved in the sand fly vectors [20,21], and viable hybrids have been achieved between geographically disparate sources of *L*. *major* [22]. Here many hybrids possess genotypes consistent with classical meiosis; however, aneuploidy with recurrent triploidy and loss of heterozygosity (LOH) was also observed. Interspecific *L*. *major/L*. *infantum* crosses have also been performed with segregation of cutaneous and visceral traits [23]. However, distinct male or female gametes of *Leishmania* have not been described, although haploid stages of *Trypanosoma brucei* have recently been discovered in tsetse flies [24].

The fact that *Leishmania* can undergo genetic exchange is of profound epidemiological importance. Genetic exchange may facilitate adaptation to new vectors, mammalian hosts or other ecological niches. For example, *Leishmania infantum/major* hybrids infect *Phlebotomus papatasi*, a non-permissive vector for *L*. *infantum* that is widespread in the Indian subcontinent [25]. Hybrids between *L*. *braziliensis* and *L*. *peruviana* have also been implicated as agents of destructive forms of mucocutaneous leishmaniasis [26]. Genetic exchange could also lead to the spread between populations of genes associated with resistance to drugs. Reassortment can potentially affect sensitivity and specificity of diagnostic methods and hybrid vigour (heterosis) could also affect virulence or transmission potential. Such implications are particularly worrying in the context of recombination contributing to the generation of novel visceralising traits in populations previously causing only dermal symptoms, or if adaptation to new vector species allows existing visceralising parasites to become more widespread.

Genome-wide sequence data are crucial to explore fully the extent of hybridisation and to identify the mechanisms by which hybrids are formed [27]. Data from only a few genetic loci may be adequate for identifying hybrids if admixture is recent or the populations have not extensively interbred. However, sparse markers are less sensitive in identifying complex, infrequent or ancient admixture, where only a small fraction of the genome may derive from any one parent. These kinds of events are known to occur in microbial eukaryote pathogens [28–31]. Parasexual recombination can also produce similar inheritance patterns and some evidence of this is seen in an experimental cross between different *Leishmania* species [23], where many progeny are also highly aneuploid. Whole-genome sequence data have been reported for only a single population of hybrid *Leishmania* from Turkey [32]. This population appears to have originated from a single hybridisation event between genetically disparate lineages within the *L*. *donovani* species complex. One of the parents appeared to be *L*. *infantum*, but the precise parentage of this population remains unclear as no putative parental genotypes were isolated in the same region. While genomic patterns were consistent with meiosis they do not formally exclude the possibility of a parasexual process.

Here we present a detailed genomic analysis of a natural hybrid population of *L*. *donovani* originating from Ethiopia. East African strains of *L*. *donovani* are particularly diverse, consisting of two main populations: one comprising strains from northern Ethiopia and Sudan, the other strains from southern Ethiopia and Kenya [33] and correspond to the areas populated by two different major sand fly vectors. *Phlebotomus orientalis* is the main vector in northern Ethiopia and Sudan and *Phlebotomus martini* in the South, although other vectors have also been implicated [34]. These two geographically (and genetically) isolated populations of *L*. *donovani* in Ethiopia also differ in clinical phenotypes [35]. High inbreeding, seemingly incompatible with strict clonality, was observed in strains from northern Ethiopia. Microsatellite [35] and MLST markers [17] have confirmed the presence of sympatric putative parental genotypes and hybrid progeny genotypes of *L*. *donovani* in isolates from the northern population. Here, we apply whole-genome sequencing data to characterise more fully these Ethiopian *L*. *donovani* to confirm that isolates are true hybrids that originate from recombination between two different sympatric lineages. We reveal a complex pattern of inheritance implying multiple independent origins from similar parents, and backcrossing with parental types. Extensive polysomy, at the level of chromosomes, is apparent in some hybrids, the significance of which is discussed.

## Results

### Genome sequencing

From each of 11 isolates, 1,600–2,300 Mb of sequence data (llumina 100 bp paired-end reads) (Table 1) were generated. When mapped against the reference genome for Ethiopian *L*. *donovani* (Isolate LV9, WHO code: MHOM/ET/67/HU3, Rogers et al. 2011) these data produced at least 40-fold median coverage across the isolates. Generally, coverage was consistent across the genome, with more than 30 reads per sample covering at least 90% of the genome (Table 1). SNP calling identified an average of 75,775 SNPs between each individual isolate and the reference genome, and this was relatively consistent across the panel, varying between 63,042 and 89,636 (Table 1). In contrast to this consistency in the number of variable sites, the proportion inferred to be heterozygous varied considerably. Some isolates showed very low heterozygosity (for example DM256, 0.015) while for others almost half of variant sites were inferred to be heterozygous (for example DM62, 0.468). Most isolates exhibiting low levels of heterozygosity (<0.1) were previously identified as putative parental genotypes [17], in contrast, putative hybrids showed much higher levels (>0.3); with the exception of one isolate (DM481) previously described as a parental type that has relatively high heterozygosity (0.267). In terms of large (>100 bp) structural variants, we observed 368 deletions, 282 inversions, 169 duplications and 264 translocations. However, many of these variants do not segregate among the recent Ethiopian isolates sequenced here (see S1 Fig), with most being heterozygous in all or most of these isolates (S1 Table, S1 Fig). A single 18 bp homozygous insertion on chromosome Ld33 was present in all of the Ethiopian isolates sequenced here, which was also present in reference strains LV9 and JPCM5 but not present in BPK282.

**Table 1:** Sequencing data and summary statistics of read mapping and variant calls. The third column indicates the classification status of the isolates suggested by Gelanew et al. 2014, rather than our conclusions based on the whole-genome data presented here. *DM19 is heterozygous at only a subset of microsatellite and MLST markers, and was described as most likely to be the result of an independent hybridization event.

isolate	WHO name	Previous classification (Gelanew et al, 2014)	Sequence reads (total Mb)	Median mapped coverage	Proportion of genome with > = 30x coverage	# of variable sites against LV9	Hetero- zygosity
DM19	MHOM/ET/2007/DM19	Possible hybrid*	21383530 (2138.3)	53	0.97	80930	0.301
DM20	MHOM/ET/2007/DM20	parental type A	19098154 (1909.8)	49	0.96	74749	0.024
DM62	MHOM/ET/2007/DM62	hybrid	19577434 (1957.7)	45	0.94	89636	0.468
DM256	MHOM/ET/2008/DM256	parental type B	19854188 (1985.4)	51	0.96	63042	0.015
DM257	MHOM/ET/2008/DM257	parental type B	23066268 (2306.6)	59	0.97	63088	0.016
DM259	MHOM/ET/2008/DM259	parental type B	16023002 (1602.3)	42	0.91	63209	0.028
DM295	MHOM/ET/2008/DM295	hybrid	20010052 (2001.0)	51	0.96	87546	0.388
DM297	MHOM/ET/2008/DM297	parental type A	20651456 (2065.1)	52	0.96	76530	0.072
DM299	MHOM/ET/2008/DM299	hybrid	22442162 (2244.2)	53	0.97	88605	0.462
DM481	MHOM/ET/2009/DM481	parental type B	20625354 (2062.5)	53	0.97	82728	0.267
DM559	MHOM/ET/2009/DM559	parental type B	18753496 (1875.3)	51	0.96	63469	0.030

### Genome-wide variation identifies distinct subgroups with different levels of heterozygosity

**Fig 1:**
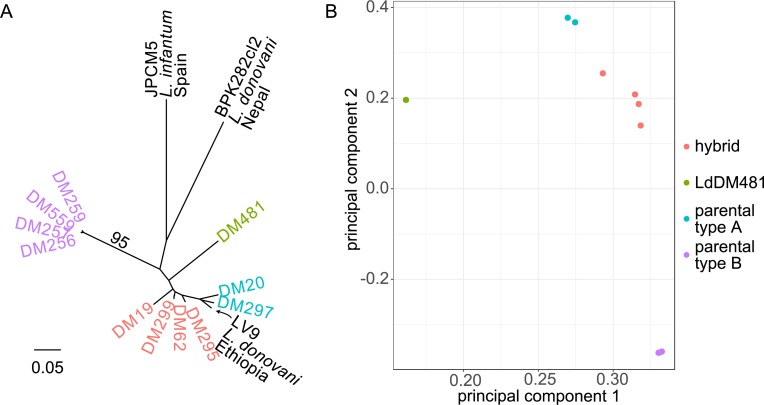
Genetic relationships between Ethiopian *L*. *donovani* isolates. Neighbour-joining phylogeny (A) and principal components analysis (PCA; B) based on genome-wide SNP variation data among Ethiopian *L*. *donovani* isolates, including additional isolates from the *L*. *donovani* species complex. Scale bar on (A) represents genetic distances in terms of expected substitutions per nucleotide site. Colors indicate groups of isolates of different putative evolutionary history as discussed in the text.

A phylogenetic tree and principal components analysis of the SNP data suggested that these 11 isolates are divided into multiple groups, with two parental groups and genetically distinct intermediate isolates (Fig 1). As expected the two parental groups are composed of isolates with low heterozygosity. The first parental group comprises DM259, DM559, DM257, DM256 and the second, comprises DM20, DM297. DM481 is an outlier (labeled in green in subsequent figures) in that it is a heterozygous sample that forms a distinct lineage, intermediate between the two parental groups but clearly distant from the other four putative hybrid isolates (DM19, DM62, DM295, DM299). Two isolates (DM62 and DM299) isolated from the same patient (a post treatment recrudescence in an HIV patient) appear very similar on the tree and PCA. The first two principal components (PCs; Fig 1B) explain 86.32% of the variance (60.32% and 20% respectively) in the data broadly reflecting previous interpretation of these isolates as hybrids and parental genotypes, the putative hybrid isolates being intermediate between the sets of parentals. An interesting exception is DM481, which appears distinct from all other samples regarding the first PC, with all subsequent PCs showing similar patterns, up to PC5, where DM19 appears as distinct from the other isolates: however, this axis encompasses only 1.9% of the total variation in these data. In the phylogeny, inclusion of three additional reference genomes (LV9, an *L*. *donovani* isolate from an Ethiopian VL patient; JPCM5, a Spanish canine *L*. *infantum*, and BPK282 from a Nepalese VL case) revealed the diversity present in this Ethiopian cohort and their distant relationship to both *L*. *donovani* in the Indian subcontinent and *L*. *infantum* (Fig 1A). The reference isolate LV9, originally isolated in 1967, appears to be closely related to the ‘parent A’ population isolates DM20 and DM297.

**Fig 2:**
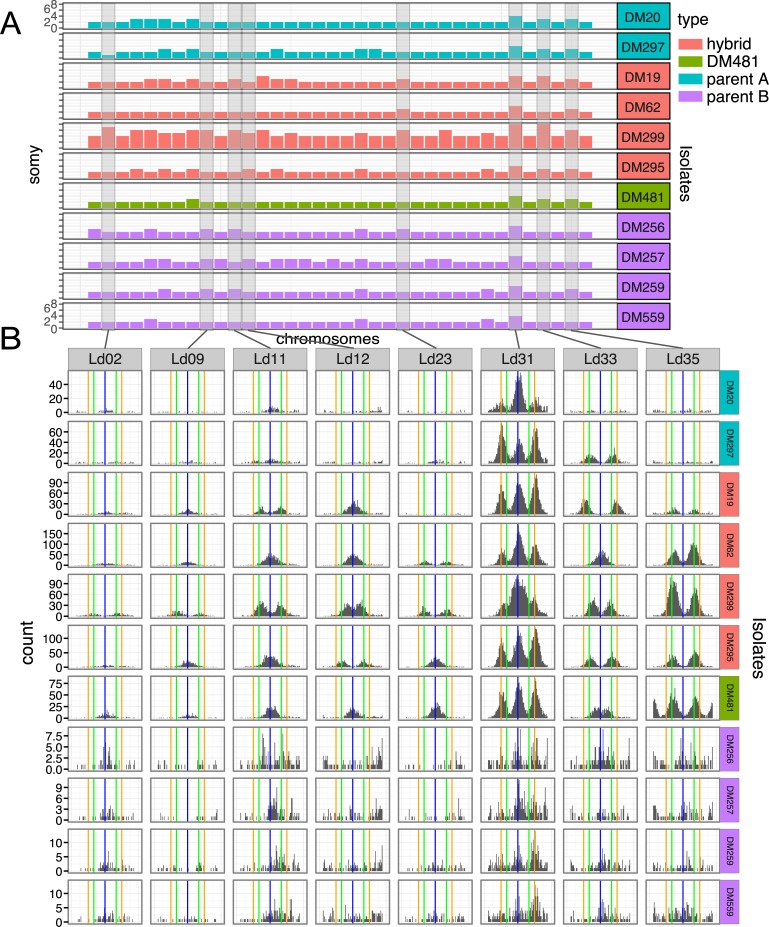
Variable somy across Ethiopian isolates inferred from coverage and allele frequencies. (A) Shows the inferred chromosome copy number (somy) for each chromosome across Ethiopian isolates under study. Y-axis scales are the same across all panel A rows. As detailed in methods, relative somy is inferred from the coverage depth of reads mapped to each chromosome, while the baseline somy is determined from the allele frequency distribution. (B) Shows distributions of non-reference allele frequencies for example chromosomes (Ld02, Ld09, Ld11, Ld12, Ld23, Ld31, Ld33 and Ld35) for each isolate, highlighting differences in somy. Vertical lines are at allele frequencies of 0.5 (blue), 0.33 and 0.67 (green), 0.25 and 0.75 (orange); expected for disomic, trisomic and tetrasomic chromosomes respectively.

Chromosome copy number for each isolate was inferred from read depth and allele frequencies at heterozygous sites (see methods, Fig 2). Most chromosomes across the majority of isolates were inferred to be diploid. However, there were notable exceptions. Chromosome 31, which, as usual in *Leishmania*, was inferred to be highly polysomic with at least four copies present in all isolates. Chromosomes 5, 6, 8, 20 and 35 were also observed at higher dosage, being at least trisomic in 6 of the 11 isolates. Two samples stood out as being more highly polysomic than others: LdDM19 was inferred to be tetrasomic at three chromosomes (13, 31 and 3), while DM299 was strikingly polysomic. For this isolate, allele frequency data suggested a minimum of tetrasomy across chromosomes, with half the chromosomes inferred at even higher dosage (6 pentasomic, 9 hexasomic, 1 heptasomic, with chromosomes 31 and 33 octasomic).

The high somy of DM299 is of particular interest given that DM62, the pre-treatment sample from the same HIV infected patient, has somy similar to the other hybrid isolates. These two isolates are otherwise genetically very similar, differing at only 4,484 sites across the genome (DM62 differs from the other hybrid isolates at 38,023 and 25,765 sites). The difference in allele frequency distribution between DM62 and DM299 is clear: for example, chromosomes 11, 12 and 33 all show a clear peak in allele frequencies close to 0.5 in DM62 suggesting disomy (or at least an even chromosome dosage), but peaks at 0.33 and 0.67 in DM299, suggesting a higher dosage of at least 3 copies (Fig 2B). The very high somy of other chromosomes is then inferred from the ratio of coverage (Fig 2A). We attempted to confirm the high ploidy of DM299 using flow cytometry to measure DNA content. Cells from the same population of cells that was sequenced were not available, but a cloned population separated from the sequenced cells by more than 8 *in vitro* passages was analysed. DNA content of these cells was suggestive of diploidy (S1 Fig), leaving some uncertainty about the precise somy of the DM299 isolate. During SNP-calling, the copy number of individual chromosomes is specified. We thus confirmed that our main results are insensitive to the assumed somy of the isolates by repeating most analyses with genotypes called as though all isolates are diploid: in all cases the conclusions from our analyses are qualitatively the same with diploid genotypes, or genotypes called using the inferred somies.

### Patterns of inheritance from putative parental populations

**Fig 3:**
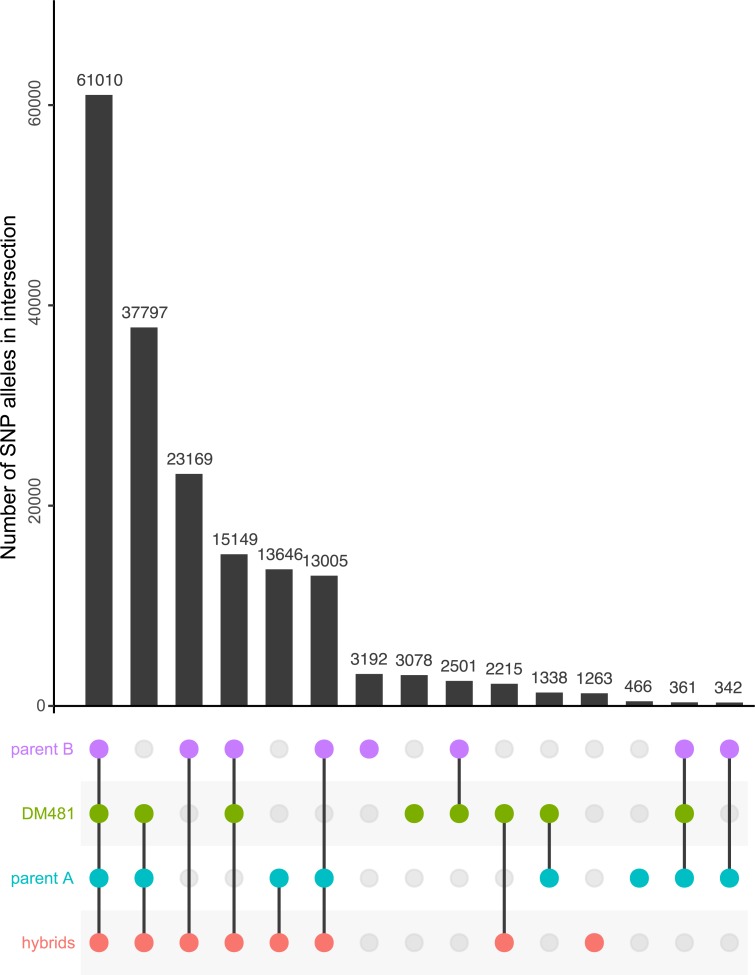
Pattern of allele sharing between groups of Ethiopian *L*. *donovani* isolates. Each row in the lower panel corresponds to one of the groups of isolates shown in Fig 1, and colored identically to that figure. Each column corresponds to a particular group or combination of groups, indicated by the colored circles present in the column. The bar graph depicts the number of non-reference alleles unique to the relevant set for that column. This figure shows an UpSet plot showing similar information to a Venn diagram in a more compact and readable format, and each column corresponds to one segment of a Venn diagram. For example, the first column represents alleles present in all four groups, and the final column alleles present in both parental groups but absent from the hybrids and DM481.

Model-based clustering is a standard approach to understanding the genetic structure of populations [36]. However, admixture or related software assumes that individuals samples derived recently from a small number of discrete, panmictic sexual populations [36], assumptions that are unlikely to be even approximately met in *Leishmania* populations. Indeed, when we applied this approach to our data, neither the recommended cross-validation nor examination of how the likelihood of the data changed with different assumptions about the number of ancestral populations (*K*) revealed a clear optimum value for the number of ancestral populations for our data (S2A and S2B Fig). With these caveats, choosing a local optimal value of *K* = 2 supported a scenario in which the proposed ‘hybrid’ isolates were mixtures of two ancestral parental populations (S2C Fig). Accordingly, variants at many sites were shared by putatively hybrid isolates and either one or other of the parental group; with relatively few variants present only in the hybrids and not found elsewhere (Fig 3). DM481 was again an exception, possessing a moderately high number of private variants and also sharing different variants with the parental groups compared to other hybrids, particularly with parent B where other hybrid isolates shared substantially more variation.

**Fig 4:**
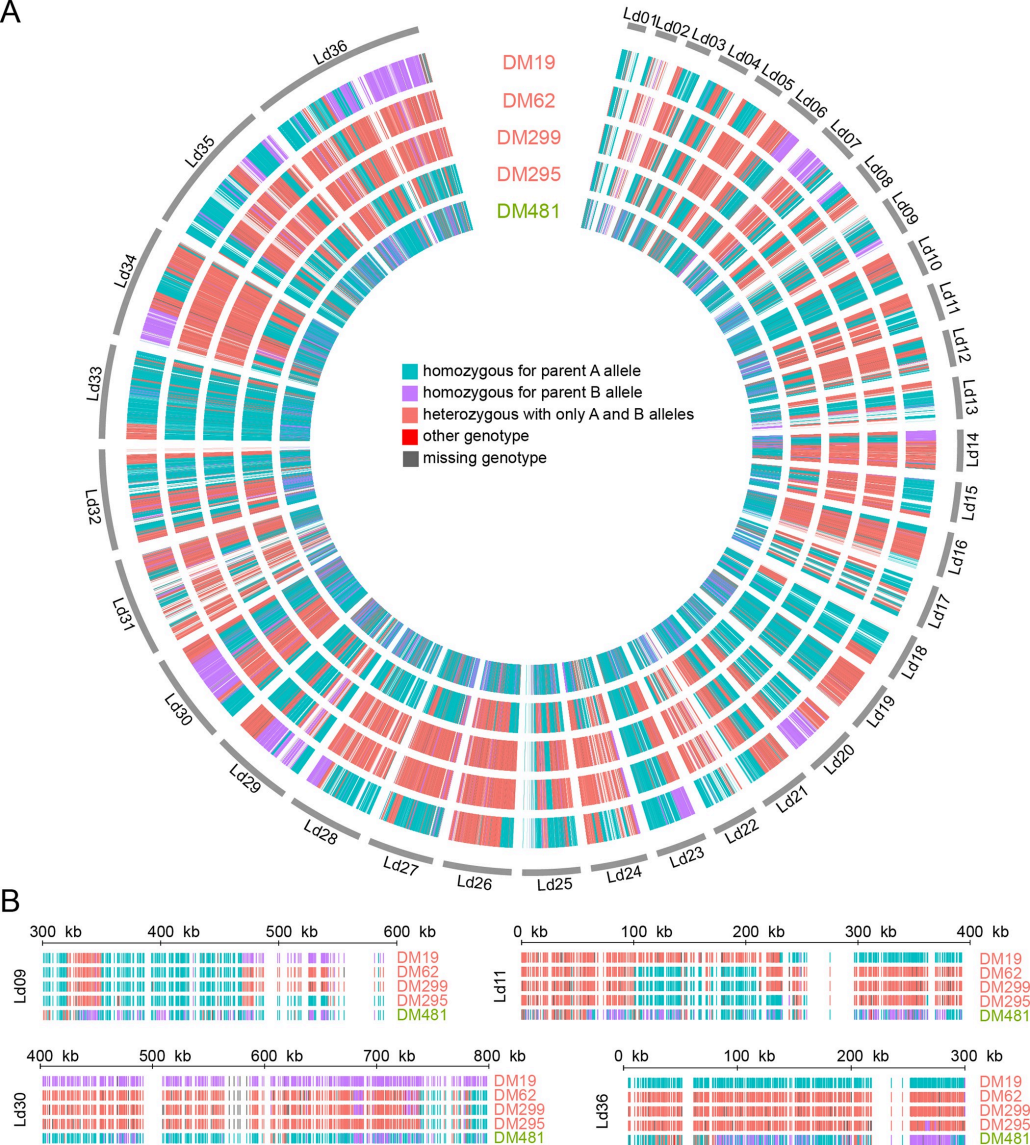
Distribution of SNPs distinguishing between potential parents. (A) Colored bars in concentric rings represent every site for which a particular SNP is unique to either of the two sets of putative parents and fixed homozygous in those parents. Colors indicate whether the variant is present as homozygous for either parental type or heterozygous with one of each allele. A small number of sites had genotypes not fixed in either parent group or no reliable genotype call. There are very few SNPs in the red or grey categories. (B) Shows a magnified view of the same data for four regions, chosen to highlight variation between different isolates.

To understand further the origins of the hybrid parasites, we identified SNP variants that are fixed differences between the two groups of parental parasites (excluding DM481) and used these as markers to identify the likely origin of variants identified in the hybrids, effectively ‘painting’ the hybrid isolate chromosomes by their likely ancestry under the hypothesis that these isolates originated as hybrids between the parental groups or close relatives. We identified a total of 49,835 such ‘parent-distinguishing SNP’ sites at which the two parental populations were completely fixed for different alleles. These sites are distributed across all chromosomes. For the vast majority of sites, across all putative hybrid isolates, genotypes consisted of a combination of these parental alleles (Table 2), supporting the notion that these isolates originated as hybrids between two parental types. The different categories of sites are strikingly unevenly distributed across the genomes suggestive of genome wide hybridisation (Fig 4). For some chromosomes these SNP markers appear to be uniformly inherited from a single parent or show uniform heterozygosity with one allele from each parental type. Most chromosomes, however, show multiple blocks of sequential SNPs of different origins, revealing a patchwork of blocks of different ancestries. Isolate DM481 is an exception: the “blocky” structure visible in other isolates is not apparent, there are less than half the number of heterozygous sites of shared origin than in any other putatively hybrid isolates, and approximately five-fold more sites in this isolate have alleles different to those fixed in the parental groups, although this still represented only 54 sites.

**Table 2:** Distributions of parental-distinguishing SNPs in putative hybrid isolates and DM481. Values are numbers of sites in each isolate from each category. For polysomic chromosomes, sites with at least one allele from each parent are grouped as ‘heterozygous AB’ irrespective of dosage of A and B alleles.

isolate	Homozygous parent A	Homozygous parent B	Heterozygous AB	Genotype not unambiguously inferred from reads	Other genotype
DM19	21,171	10,419	16,633	1,602	10
DM62	15,600	2,103	29,779	2,343	10
DM295	25,482	1,429	21,032	1,880	12
DM299	15,868	2,036	18,571	13,352	8
DM481	31,104	10,271	7,400	1,006	54

**Fig 5:**
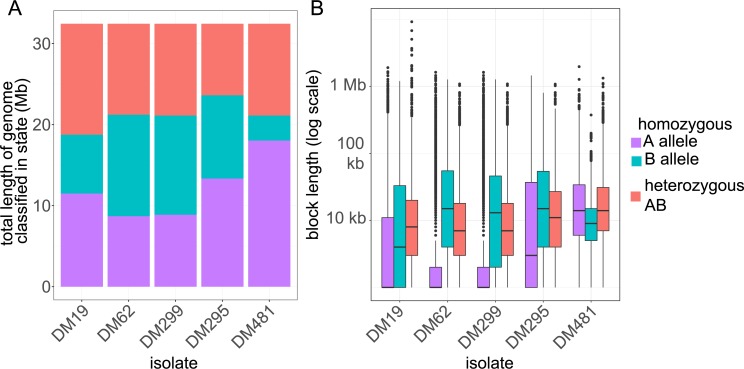
Distribution of genomic regions of putative hybrid isolates between parental origins based on Hidden Markov Model. (A) Shows the total number of basepairs assigned as homozygous parent A, homozygous parent B and heterozygous based on the maximum posterior probability assignment of hidden states of the Hidden Markov Model. (B) Box-and-whisker plot showing the distribution of lengths of contiguous blocks assigned to each of these three parentage states across the 5 putative hybrid strains in the most probable path identified in the Viterbi decoding. Boxes show median length and interquartile range on a log axis, whiskers represent the range of data excluding outliers (defined as those outside 1.5 times the interquartile range).

To get a more quantitative understanding of the distribution of these parent distinguishing SNP sites in blocks inherited from each parent we developed a hidden Markov model (HMM) to assign putative ancestry for sites without parent-distinguishing SNPs. The HMM assigns every 100 bp window of the genome to three states: either homozygous ‘parent A’, homozygous ‘parent B’ or heterozygous (Fig 5A). The advantage of the HMM is that it can statistically assign windows even in the absence of any parent-distinguishing variants (or where those variants are ambiguous), under the assumption that transitions between the states occur in a regular way across the genome if there is no direct evidence of a change. The HMM assigns significantly different proportions of the genome to each category for different isolates (Fig 5A), and the uneven distribution of sites in each category across the genome is reflected in the fact that these proportions are quite different from the proportion of parent-distinguishing SNP sites assigned to each category. This model also estimates the length of ‘runs’ that form blocks of genome with a single inheritance pattern. The distribution of these block lengths provides information on the relative age of hybrids, as we would expect recombination to have broken down blocks from older introgression events more than recent events. Longer blocks suggest that fewer hybridisation events have occurred between admixed clones but backcrossing to parental clones or related parasites would also contribute to longer blocks. The block length distribution varies between isolates (Fig 5B): it suggests that DM62 and DM299 represent a more recent hybridisation with a ‘parent B’ type than any ‘parent A’ type, and that DM295 may originate from a more recent hybrid (or with fewer hybridisations) between the two parental types than DM19. For at least two isolates (DM62 and DM299) the inheritance is asymmetrical, in that they are inferred to have inherited different proportions of their genome from each of the parental types, suggesting that these isolates did not originate by crossing within a ‘founder’ hybrid population, but involved some degree of backcrossing with parent B types.

### Reconstructing haplotypes confirms that variants of each parental type are linked

**Fig 6:**
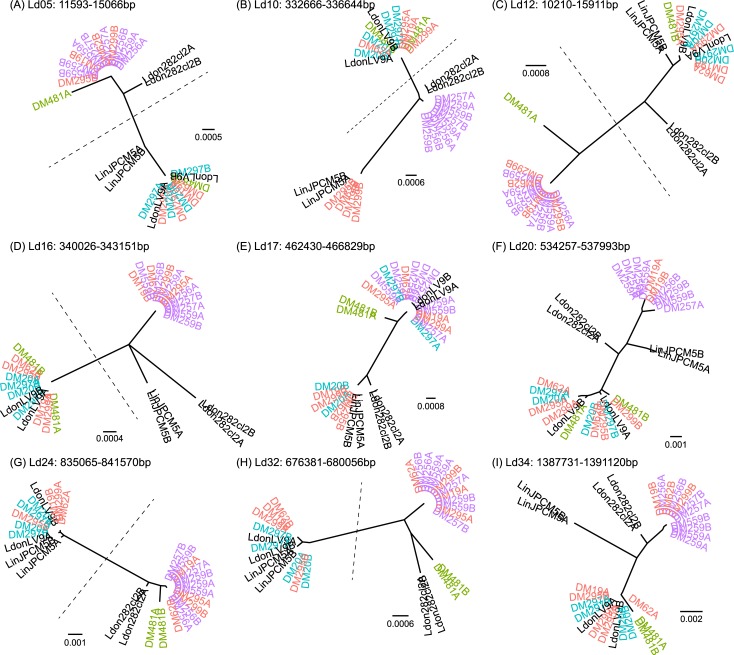
Phylogenies of inferred haplotypes for Ethiopian *L*. *donovani* isolates. Shown are maximum-likelihood phylogenies for inferred haplotypes at the 9 genome regions at which all isolates could be phased into blocks of length greater than 3kb and with an average of at least 4 heterozygous sites per isolate. A and B labels on the leaves are arbitrary names for the two different haplotypes at each locus, for each isolate. Dotted lines separate the two hybrid haplotypes for those blocks at which all the hybrid isolates (not including DM481) have one haplotype from each putative parental population. Isolates are colored as in Fig 1, and titles indicate the positions of these haplotypes on the reference genome assembly. Panels A-I each show the phylogeny for a different block, with genome co-ordinates as shown on the figure.

We used reads and read pairs to phase locally heterozygous sites that were physically close to each other in the genome into sets of variants known to be present on a single haplotype. We subsequently identified regions at which all heterozygous positions were phased in all 15 isolates, so that we have unambiguous information about the haplotypes present in these regions. We inferred haplotype phylogenies for nine such regions that were at least 3 kb long and had an average of at least 4 heterozygous sites per isolate; this included 9 of the 16 ‘fully phased’ regions of 3 kb or longer (Fig 6). All 9 blocks were on different chromosomes.

In most blocks (7 out of 9; Fig 6A–6E, 6G and 6H) the two haplotypes for each of the four hybrid isolates (DM19, DM62, DM295 and DM299) cluster in different parts of the phylogeny. For 6 of these (Fig 6A–6D, 6G and 6H), phylogenies show the expected pattern if the hybrid isolates originated from a single hybridisation between the two parental types: a long branch of the haplotype tree separates all parent A haplotypes together with one haplotype of each of the 4 hybrids from parent B haplotypes with the second haplotype of each hybrid. In one block (Fig 6E) on chromosome 17 the two haplotypes for each hybrid isolate divide into two clusters as expected, but the two ‘parent A’ isolates (DM20 and DM297) appear in different clusters. In the chromosome 34 block, both haplotypes for one hybrid isolate (DM295) clustered with the same parental group–the parent A isolates (Fig 6I). All of these haplotypes are consistent with a hybrid origin for the isolates but with a more complex history than a simple, single hybridisation between the two parental populations. This could involve further recombination either by ‘intercrossing’ within the hybrid population or backcrossing to the parental types.

**Fig 7:**
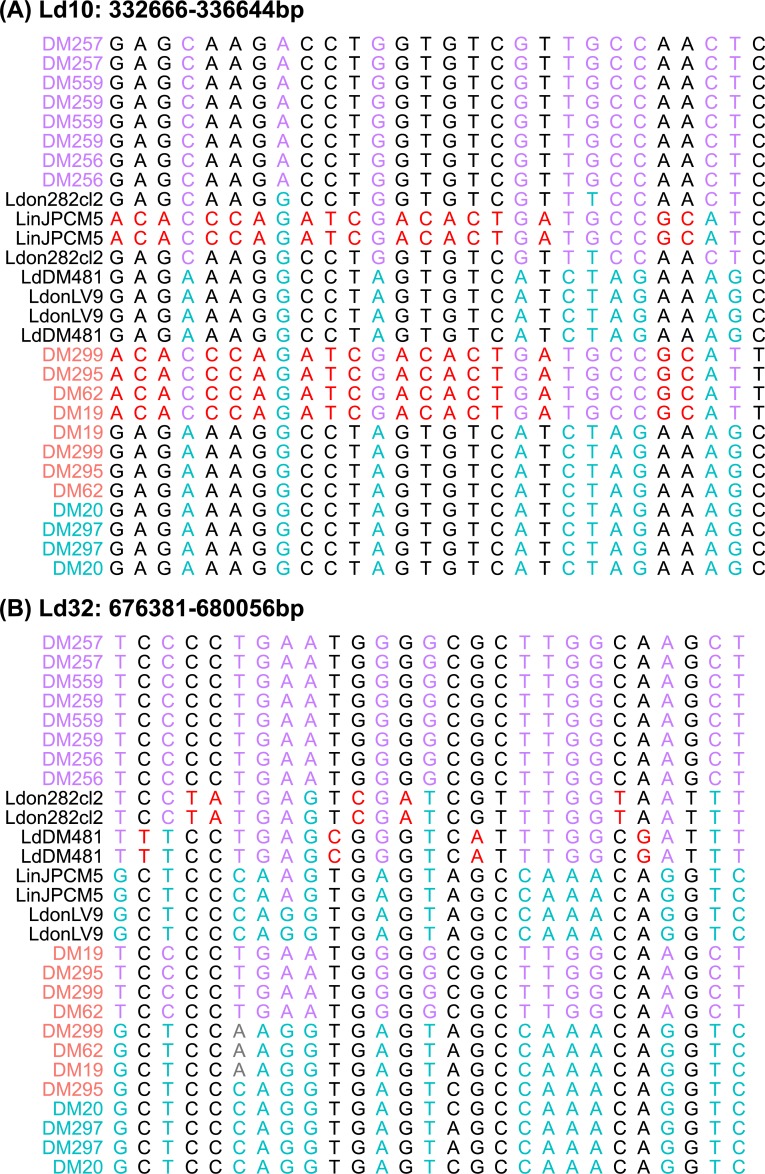
Unusual haplotypes in phased regions of Ethiopian *L*. *donovani* isolates. Alignments of inferred haplotypes for fully-phased regions of Ethiopian *L*. *donovani* isolates, showing unusual haplotypes in (A) the hybrid isolates and (B) DM481. Panels show all variable sites within two of the ‘fully phased’ genome regions shown on Fig 6, and titles indicate the positions of these haplotypes on the reference genome assembly. In all panels, red sites identify alleles specific to two unusual haplotypes discussed in the text. Cyan and magenta identify sites at which parent A and parent B isolates share fixed different alleles (parent distinguishing sites). Isolates are colored as in Fig 1.

Only the final block (Fig 6F) does not support a simple hybrid origin for these isolates, as the two haplotypes for each isolate cluster together. Further rounds of crossing, with a different history for LdDM19 and the other hybrid isolate, would explain the pattern at this locus. Examining the alignments for these blocks did not reveal any sign of recombination within reconstructed haplotypes, but did reveal some haplotypes in the putative hybrids that differ from either of the putative parental types—for example at the haplotype block on chromosome Ld10 all of the hybrids share one haplotype that is very similar to those in *L*. *infantum* JPCM5 but missing from any of the other *L*. *donovani* isolates (Fig 6B; Fig 7A). Either the parent B isolates are a poor proxy for the true parental types at this locus, or the history of the hybrid isolates includes crossing with more than two parental populations.

The pattern for DM481 haplotypes was more complex: in 7 out of 9 trees the two haplotypes for this isolate appear in the same cluster: twice with parent B haplotypes (Fig 6G and 6H) although never very closely related to these; four times with parent A haplotypes (Fig 6B, 6D, 6F and 6I). In one other case (Fig 6E) the parent A isolates are themselves non-monophyletic. At many of these loci, DM481 has haplotypes not present in other isolates. At two other loci (on Ld05 and Ld12; Fig 6A and 6C) DM481 is heterozygous for one haplotype not observed elsewhere and for one haplotype shared with parent A isolates and the hybrids. At the phased locus on Ld32 (Fig 6H), the DM481 haplotype is apparently distantly related to BPK282; although closer inspection of this locus shows they are united by their lack of alleles present in other isolates rather than shared characters (Fig 7B).

For the other “outgroup” isolates of *L*. *infantum* and *L*. *donovani*, the two haplotypes from each isolate consistently cluster together. Wherever parent A haplotypes are monophyletic, the Ethiopian LV9 isolate haplotypes group with them. Nepalese *L*. *donovani* BPK282 tends to group with the parent B isolates but is often clearly distinguishable from them; the position of *L*. *infantum* JPCM5 haplotypes on these trees is more variable.

### Kinetoplast (kDNA) phylogeny

**Fig 8:**
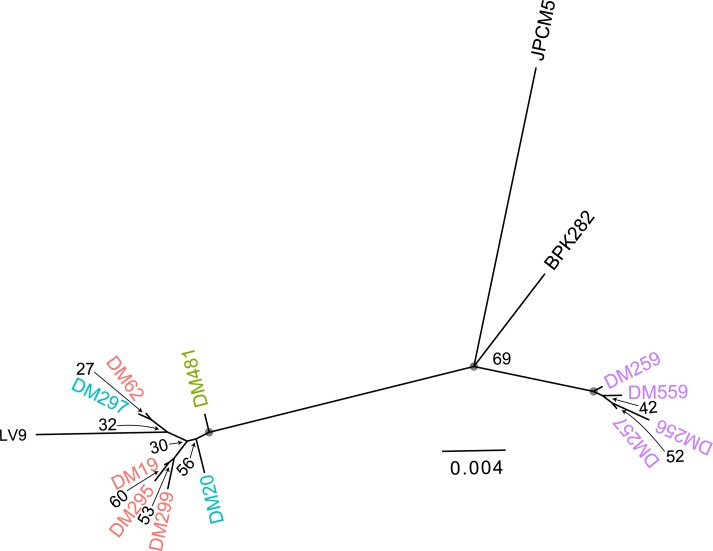
kDNA phylogeny of Ethiopian *L*. *donovani* isolates. Maximum-likelihood phylogeny of reconstructed mitochondrial (kDNA maxicircle) haplotypes for Ethiopian *L*. *donovani* isolates, Values on nodes are bootstrap support values for the partition induced by deleting the edge below each node, grey circles denote 100% support. Isolate color labels are as in Fig 1.

The kDNA maxicircle is homologous to the mitochondrial genome of other eukaryotic groups [37], and is thought to be uniparentally inherited in *Leishmania* [20] and trypanosomes [38]. The maxicircle phylogeny (Fig 8) shows a close relationship between the hybrids, parental type A, DM481, and the historical reference LV9 isolate. These are phylogenetically distinct from parental B isolates. This contrasts with the nuclear phylogeny, which shows the hybrid samples as somewhat more closely related to parent A isolates but clearly intermediate between both parental groups: here, the parental A isolates do not even form a monophyletic group, with LV9 and hybrid DM297 clustering with one parental type. Surprisingly, the mitochondrial phylogeny suggests some divergence between DM299 and DM62, despite them originating pre and post treatment from the same patient. However, supporting bootstrap values were low for all relationships in this part of the tree. The outlier isolate DM481 also appears less distant from the cluster comprising parental group A and hybrids than indicated from nuclear SNP variation, even though mutation rates are likely to be an order of magnitude greater for mitochondrial data than for the nuclear genome. The uncertainty in the precise relationships between isolates notwithstanding, the close relation between parental group A and the hybrid isolates suggests the hybrids uniparentally inherited parent type A mitochondrial genomes, and gives some support to the idea that these all originated from a single initial cross, although these data cannot exclude that there is some bias in the inheritance of kDNA between strains, or that this shared kDNA type reflect subsequent backcrossing rather than the original hybridisation.

## Discussion

Hybridisation in *Leishmania* has now been demonstrated experimentally and is also observed in natural isolates across different species. These include multiple *L*. *braziliensis-L*. *peruviana* hybrids in Peru [26], *L*. *infantum-L*. *major* hybrids [25] and a widespread lineage of *L*. *tropica* that appears to be disseminated from a recent hybridization event [15]. However, the only previous whole genome analysis of hybrid *Leishmania* isolates identified a population from Turkey that appeared to be hybrids between a MON-1 genotype type of *L*. *infantum* and another member of the *L*. *donovani* species complex [32]. In that case, isolates appear to be generated solely from continued crossing within an initial hybrid population without back-crossing to either parental type. The genetic heritage of Ethiopian hybrids we describe here must be more complex. More specifically we propose that these isolates must have originated from more than one crossing event between similar parents. Additionally, crosses must have occurred between either different hybrid types or between hybrids and parentals subsequent to the ‘founding’ outcrossing event.

In more detail, our results are indicative of complex *L*. *donovani* populations in northern Ethiopia, We find two distinct parental groups of isolates with low heterozygosities. The first parental group, comprising DM259, DM559, DM257, DM256 and the second, comprising DM20, DM297. We confirm that four of the heterozygous isolates (DM19, DM62, DM295 and DM299) possess hybrid genotypes that are phylogenetically intermediate between these two parental groups. On both the tree and PCA, these are not equidistant from the two parental groups, suggesting a greater contribution of the second group of parental isolates (parent B) to these putative hybrid genotypes. Admixture analysis supports the interpretation of these intermediate genotypes as hybrids. Most blocks of variants in these genomes can be interpreted as being inherited from one, or both of the parental populations. The large-scale structure of these genomes as blocks of variants of particular ancestry suggests that these are relatively recent events, at least in terms of the number of crossing-over events that have occurred since. There are however substantial differences between the hybrids in that the number of variants shared by individual hybrid isolates and the parentals, and also their distribution across the genomes differs substantially. The structural variation we identify does not appear to support a hybridisation scenario, but the many shared variants probably represent differences between both Ethiopian populations and the reference genome assembly rather than genuine variation within these populations. It is clear that structural variation appears rapidly during in vitro culture of *Leishmania*, so we cannot be sure that all of the variants we detect are present in natural populations rather than being an artefact of promastigote culture [39–41]. In the absence of gold standard known structural variation or any experimental validation it is possible our variant calls themselves are quite noisy.

Results are indicative of at least three different histories of crossing between parental types, and probably within the hybrid population itself. Here with DM299 and DM62 (isolated from the same patient) showing very similar patterns probably arising from the same isolate, while both DM19 and DM295 are distinct with different histories. Using a hidden Markov model, we estimated the lengths of blocks of sequence inferred to originate from each parental population. The distribution of sizes of contiguous sequence fragments derived from each parent was not consistent between the hybrids, probably reflecting differences in the timing of hybridization events; we expect that continued interbreeding and the accompanying crossing-over gradually broke down these blocks of ancestry. Hence, blocks inherited from parents in older crossing events have smaller stretches of continuity. Our HMM results thus gives qualitative insight into the relative age of the different events that gave rise to these hybrid isolates. Using this approach, we infer that DM62 and DM299 have crossed onto a parent B like ancestor more recently than to parent A, while the ancestry of DM19 and DM295 from both parents is approximately equally old. This approach of assessing length of inherited haplotypes has been previously used to gain quantitative results into the history of human populations [42], but parameterising this kind of approach in *Leishmania* seems challenging, given the facultative nature of sexuality in this genus, and our current lack of understanding of recombination in *Leishmania*. In particular, we do not possess quantitative information on the mutation rate, recombination rate or even likely generation time of *Leishmania* in vivo. In all cases a greater proportion of the genomic variation was shared with the ‘parent A’ population, suggesting a more recent common ancestry with this population or backcrossing with this population during evolutionary history. The close relatedness between the LV9 isolate, isolated in the Humera district of Ethiopia in 1967, and one of the parental groups indicates that parental like genotypes have been present in this region for extended periods of time. While the age of the hybridisation events we have described are unclear, the presence of genotypically stable “parental donors” over time may have facilitated the emergence of multiple hybrid populations. In summary, while we cannot reconstruct the precise history of this population, our data confirm that the population of *L*. *donovani* in Ethiopia has undergone multiple rounds of hybridisation, including more complex patterns of crossing than simple F1 hybridisation between parents or subsequent crossing within a hybrid population.

Isolate DM481 emerged as a consistent outlier that forms a distinct lineage, intermediate between the two parental groups but also distinct from the other four putative hybrid isolates. The presence of haplotypes at a number of loci in this isolate which are not present in either parental population makes it seem unlikely that this is a very recent hybrid between the two parental groups in the current cohort. One explanation is that DM481 is simply a representative of a distinct, divergent population, which appears plausible considering the genetic diversity within *L*. *donovani* that has previously been identified within this region [17,33,35]. A more unlikely scenario is that DM481 has some recent common ancestry with the parent A population but less so with population B.

The multiplicity of hybrid and parental genotypes within this small sample of isolates from northern Ethiopia suggests that genetic exchange is commonplace among *L*. *donovani* populations transmitted by *P*. *orientalis*, and that resulting hybrid progeny may be widely disseminated. This complex evolution also implies that co-infection of *P*. *orientalis* with different *L*. *donovani* isolates occurs frequently, at least in this region. It is currently unknown if hybrids are most likely to emerge when a sand fly is co-infected with different *Leishmania* strains ingested in the same blood meal or subsequent feeds. For *T*. *brucei* there is evidence that the production of hybrid genotypes is most successful when both parental types are taken up in a single meal by the tsetse vector [43].

The pattern of polysomy we observe across the cohort did not reflect the phylogenetic relationship between isolates or their assignment to hybrid or parental classes; this is consistent with a highly dynamic chromosome complement in *Leishmania* promastigotes described both experimentally [44] and in field samples, for example in *L*. *donovani* in the Indian subcontinent. Indeed, aneuploidy patterns do not seem to strongly segregate between Ethiopian *L*. *donovani* populations [33] despite the degree of nucleotide diversity identified in these samples from one region of Ethiopia alone being much higher than in the ISC. For example, pairs of samples in the main ISC population differ on average at only 88.3 sites whereas in the current cohort even two of the closely related ‘parent B’ isolates vary at an average of 1038 sites. Aneuploidy is known to be beneficial in allowing some single celled eukaryotes, for example *Saccharomyces* and *Candida*, to rapidly generate adaptive diversity [45], and likely contributes to adaptation in *Leishmania* [46,47]. Aneuploidy is known to impact gene expression in *Leishmania* promastigotes [48,49]. As *Leishmania* lacks classical regulation of transcription at initiation through promoters, this could contribute to parasite adaptation to at least some conditions [39,46]. However, it is unclear how extensive aneuploidy variation in cultured promastigotes is relevant to the situation in either natural vectors, or in amastigotes *in vitro* or *in vivo*: while it is clear at least some variation does occur in both [48,50] it appears to be much less widespread than in *in vitro* culture.

A striking result is that the sequence data suggests that isolate DM299, a recrudescent infection (from DM62) taken from the same patient was remarkably polysomic across all chromosomes relative to other isolates. Previous flow cytometry measurements of DNA content were suggestive of diploidy for both strains DM62 and DM299 and all other strains in the cohort [17] and are therefore incongruent. However, a potential confounder was that the original cloned line that was sequenced was not available for cytometric analysis, with the isolate having undergone additional passages (>8). Somy in *Leishmania* can vary dramatically and rapidly in culture [48,51] and prolonged *in vitro* culture is known to systematically reduce ploidy in experimentally derived *T*. *cruzi* [52]. Here, relative somy between chromosomes is inferred from the coverage depth of reads mapped to each chromosome, while the baseline somy is determined from the allele frequency distribution. In principle, this could be misleading if the samples sequenced were mixtures of clones with many different somy levels, and single cell approaches such as FISH or single cell sequencing would be needed to fully disentangle this [53]. However, the differences in allele frequency distributions between DM62 and DM299 for many chromosomes is particularly striking (Fig 2B), so there are at least genuine differences in the complement of chromosomes between the sequenced isolates. The consistently high dosage of some chromosomes–most strikingly chromosome 31 – are also broadly consistent with previous reports [40,48,54]. Together these provide reassurance that somy inferences are correct. We speculate that the apparent remarkable differences in somy between LdDM62 and LdDM299 isolated from the same HIV patient, could be an adaptive response to either chemotherapy or suppression of the patient’s immune response. SNP differences between DM62 and DM299 isolates were minimal, so aneuploidy variation could be a convenient mechanism to alter gene expression in response to drug pressure, as demonstrated in *Leishmania* [46] and conclusively in resistance of some pathogenic fungi to azole drugs [55,56].

Broadly, mitochondrial phylogenies corresponded to the expected nuclear genotypes (Figs 1 and 8 respectively). These data suggest that hybrids uniparentally inherited parent type A mitochondrial genomes in agreement with inheritance patterns seen previously in other trypanosomatids [57]; [58]. While all analyses support the clustering of LV9, parent A and hybrid isolates, there is little bootstrap support for the placement of different isolates within this cluster, and these placements varied with details of the mitochondrial maxicircle assembly approach. In this context, it is difficult to assess the significance of the apparent divergence between DM62 and DM299 (isolated from the same patient pre and post treatment), and we do not interpret the small differences between nuclear and mitochondrial phylogenies as evidence of mitochondrial introgression. Mitochondrial introgression would be a very specific marker of hybridisation between populations and has been described in trypanosomatids, including *T*. *cruzi* [57] and in many other organisms [59]. Different ancestries between mitochondrial and nuclear genomes would not be expected between DM62 and the recrudescent infection DM299 in that they are likely to be the product of a single hybridisation event, based on near identical genomic structure and SNP profiles.

Current understanding regarding pattern and process of hybridisation in *Leishmania* is incomplete. Analysis of populations to detect and describe genomic variation in evolutionary recent hybrid isolates can confirm that hybridisation occurs in natural populations and provides insight into rates and patterns of recombination. For example, previous estimates based on genomic analysis from natural *L*. *infantum* isolates from Turkey indicate a hybridisation frequency of 1.3 x10^-5^ meioses per mitosis [32]. However characterisation of natural systems presents particular challenges: while co-localised isolates similar to the putative parents can sometimes be found, this is not guaranteed [32]. The number of independent meioses sampled in a natural population can be small and is consistently difficult to quantify. The recent ability to derive experimental hybrids in *L*. *major* [20] and now *L*. *donovani* [21] can facilitate our understanding, as multiple replicated offspring from identical (and known) parents, frequency and distribution of cross-overs are easier to assess. Particular questions of interest are whether recombination tends to occur at particular localised hot-spots? If so, are they associated with particular genomic features such as GC content or between polycistronic transcription units? Are crossing-over events associated with particularly high SNP mutation rates [60]? In the current data we do not observe particular clusters of SNPs absent in the parental populations that could suggest this, as these data have limited power to detect these effects, which would require large number of observations of independent crossing-overs between the same parental haplotypes. Similarly, the contribution of gene conversion, often associated with meiosis, on either SNPs or tandem gene families are difficult to infer in these natural data. It is also important to note that ‘parental’ isolates represented are only proxies for the true parents but our results are strongly suggestive of multiple recombination events in Ethiopian *L*. *donovani* in recent evolutionary history. Encouragingly, experiments and subsequent derivation of experimental hybrids from phylogenetically similar parental genotypes also suggest frequent recombination in different sand fly vector species [21].

Experimental work will produce quantitative insights to support deeper understanding of the mechanisms and implications of recombination in *L*. *donovani* populations. Understanding how these mechanisms operate in natural populations will certainly require continuing work and additional data from the wild, but equally important may be the development of methods to analyse these data. Here, we present a largely qualitative look at a relatively small collection of isolates. While powerful methods to extract quantitative information about natural selection, demography and gene flow from genetic samples of natural populations exist [61][62]. these approaches rely on simplifying assumptions about the genetic process, and certainly do not capture the complexities of variable aneuploidy, combined sexual and asexual reproduction and the possibility of unusual forms of sex that are relevant to *Leishmania* populations. Another important complexity of *Leishmania* is the complex lifecycle involving population bottlenecks during vector transmission, and that *Leishmania* are present within host and vector populations, so that epidemiology will also impact their populations. The required methodological developments will be informed by baseline data such as we present here.

In conclusion we have presented genome-wide sequence data for putatively hybrid isolates of *L*. *donovani* from human VL cases in Ethiopia, together with isolates possessing putative parental like genotypes. We confirmed that 4 of the 5 putative hybrids are indeed hybrid offspring derived from strains related to these parents, but the evolutionary history of these isolates is complex: representing at least 3 different histories. The haplotypic reconstructions, distribution of parent distinguishing SNPs and patterns of allele sharing are consistent with the occurrence of more than one hybridisation event and/or intercrossing and backcrossing to parentals, which has not been observed in experimental crossing experiments to date. These data thus confirm the ability of *Leishmania* to hybridise extensively in natural populations. The population of *L*. *donovani* in Ethiopia has undergone multiple rounds of hybridisation, and we predict complex patterns of crossing would be revealed by a more substantial sample size. Together with progress in deriving experimental hybrids there is now promise of elucidating the mechanisms and other phylo-epidemiological aspects of recombination that have widespread implications regarding the spread, diagnosis and control of *L*. *donovani* populations.

## Materials and methods

We generated short-read paired-end sequence data for 11 isolates of *Leishmania donovani* from Ethiopia (see Table 1). Full details of the origin and isolation of the strains used are described elsewhere [17,35]. Briefly, all were visceral leishmania isolated between 2007 and 2009 from humans in northern Ethiopia. Of note, isolate DM299 was a relapse of DM62 isolated from a HIV infected patient post treatment (Libo Kemkem-Abdurafi).

DNA library preparation was performed by shearing genomic DNA into 400–600 base pair fragments by focused ultrasonication (Covaris Adaptive Focused Acoustics technology; AFA Inc., Woburn, USA), standard multiplex Illumina libraries were prepared using the NEBNext DNA Library Kit. The libraries were amplified with 8 cycles of PCR using Kapa HiFi DNA polymerase’ and were then pooled. 100bp paired-end reads were generated on the Illumina HiSeq 2000 v3 according to the manufacturer’s standard sequencing protocol. All sequencing data for these isolates are available from the European Nucleotide Archive under project ERP106107. The LV9 strain (MHOM/ET/67/HU3 also known as MHOM/ET/67/L82) was originally isolated from a VL case in the Humera district in the far North of Ethiopia in 1967 [63]. The JPCM5 strain (MCAN/ES/98/LLM877) is an *L*. *infantum* from Spain, isolated from a dog in 1998; BPK282 (MHOM/NP/03/BPK282/0cl2) was isolated from a human VL case in Nepal in 2003. Illumina whole-genome data for these isolates were obtained from the ENA database, with parasite material, sequencing approach and analysis of these data detailed in [40] for JPCM5 and LV9, and in [54] for BPK282.

Reads for each isolate were mapped to the *L*. *donovani* LV9 reference assembly using SMALT v0.7.0.1 [64], indexing every second 13-mer [65] and mapping repetitively with a minimum identity of 80% and maximum insert size of 1200bp, and mapping each read in the pair independently (*-x* flag). Variants were called using the HaplotypeCaller algorithm of Genome Analysis Toolkit v3.4 [65], following best-practice guidelines [66] except as detailed below. Variant calls were first filtered to remove any overlapping with a mask generated with the GEM mappability tool [67] to identify non-unique 100bp sequences and to remove 100bp either side of any gaps within scaffolds. Subsequent filtering with the Genome Analysis Toolkit removed sites using the filtering parameters: DP > = 5*ploidy, DP < = 1.75*(chromosome median read depth), FS < = 13.0 or missing, SOR < = 3.0 or missing, ReadPosRankSum < = 3.1 AND ReadPosRankSum > = -3.1, BaseQRankSum < = 3.1 AND BaseQRankSum > = -3.1, MQRankSum < = 3.1 AND MQRankSum > = -3.1, ClippingRankSum < = 3.1 AND ClippingRankSum > = -3.1. Calls were made both assuming diploid genotypes for every chromosome for each isolate, and using a somy estimated for each chromosome independently for each isolates. Somy was estimated using the EM approach described previously [49], and values checked by manual inspection of read depth and allele frequency data.

The whole-genome phylogeny and principal components analysis presented here were generated by using VCFtools v0.1.15 [68] to convert the variants from GATK vcf format to the input format for plink, and then plink v1.90b3v [69] was used for the principal components analysis and to generate pairwise distances (1—identity by similarity). The pairwise distances were used to calculate a neighbour-joining phylogeny using the neighbor program from PHYLIP v3.6.9 [70]. Phasing was based on identifying illumina reads and read pairs linking heterozygous sites within each isolate, using the phase command in samtools v.0.1.19-44428cd [71], with a block size (k) of 15; the phasing results did not differ for other values of k tested (11, 13, or 20) except k = 30, where few variants were phased and no blocks > 1kb were shared by all isolates. Note that this phasing approach identifies heterozygous sites *de novo* from read mapping data rather than using the variant calls, and reconstructs at most two haplotypes at any locus. Phylogenies for the inferred haplotypes were generated using raxmlHPC v8.2.8 [72] under a GTR+I+G model of nucleotide substitution and otherwise default parameters. Model-based clustering was performed using Admixture v1.23 [73] with a set of 23,439 variants remaining after pruning with plink v1.9 [73,74] so that no window of 20 consecutive variants contains any pairs of variants in high linkage disequilibrium R^2^ > 0.25.

Parent-distinguishing sites were identified as those for which both parent A isolates shared an identical homozygous genotype and all four parent B isolates were homozygous for a different allele. These sites could be unambiguously assigned as being derived from one or other parent in the putative hybrid isolates, assuming these other isolates were hybrids of these parents. To extend this analysis to other sites across the genome, a Hidden Markov model (HMM) was used to classify every 100bp window along the genome of the 5 suspected hybrid isolates by likely ancestry. Three hidden ancestry states (homozygous parent A, homozygous parent B and heterozygous from each parent) were used to explain the pattern across the genome of 4 observed parent-distinguishing SNP “symbols” (homozygous A, homozygous B, heterozygous and a non-determinate symbol for windows with either no parent-distinguishing SNPs or more than one state). The 100bp window size was chosen to make the HMM computationally tractable and so that almost every window (198,679 out of 202,940 across 5 isolates) was unambiguous for the observed symbol. All transitions between hidden states were allowed, but each hidden state could emit only the corresponding observation or the non-determinate symbol. Initial transition and emission probabilities and trained parameters are shown in S2 Table; the trained parameters did not depend strongly on the initial parameters. The HMM was trained independently on each chromosome and isolate, and then average transition and emission parameters, weighted by chromosome lengths used to infer hidden states. Training and Viterbi decoding of the HMM was performed using the HMM package in R v3.3.0 [75].

kDNA maxicircle genome sequences were generated by mapping illumina sequence data against the available maxicircle sequence assembly for *L*. *tarentolae* [76] and using MITObim [77] to perform iterative guided assembly with block size (k parameter) of 61 and with read trimming. This produced assemblies of between 19,611bp and 21,682bp in a single contig in each isolate (the *L*. *tarentolae* maxicircle is 20,992bp), including the entire transcribed region: tests using less strict criteria for assembly produced longer but less reliable assemblies. The assembled contigs were then rotated using CSA [78] before aligning with MAFFT v7.205 [79] with automated algorithm choice (—automated1 flag); the alignment was then trimmed with trimAl v1.4 [78,80]. A maximum-likelihood phylogeny was inferred using raxmlHPC v8.2.8 under a GTR+I+G model of nucleotide substitutions [72] with 10 random addition-sequence replicates, and confidence in branches of the tree assessed with 500 bootstrap replicates.

## Supporting information

S1 Table Large (> 100bp) Structural variation between isolates (* this does not include LV9). (DOCX)

S2 Table Initial and trained parameters of HMM. Initial transition (a) and emission (b) probability matrix and trained transition (c) and emission (d) probabilities for HMM. NA represents “Not Allowed” emissions from that state.
(DOCX)

S1 Fig Structural variation between isolates. White bars represent homozygous reference genotypes, dark grey homozygous non-reference genotypes and the intermediate shade heterozygous positions. Purple bars represent low-quality calls where genotype is uncertain.
(EPS)

S2 Fig Model-based clustering with Admixture. (A) Five-fold cross-validation error and (B) Likelihood of the data for models with different numbers of ancestral populations (*k*). (C) Maximum-likelihood ancestry proportions for each isolate under a model with k = 2. Colors of bars represent two different hypothetical ancestral populations, colors of isolate labels on X-axis represent parental and proposed hybrid populations of isolate, as shown in Fig 1.
(EPS)
