# Supplementary material for: Genomic analysis of natural intra-specific hybrids among Ethiopian isolates of Leishmania donovani
Source: PLoS Negl Trop Dis. 2020 Apr 20;14(4):e0007143. doi: 10.1371/journal.pntd.0007143 (PMC7237039; doi:10.1371/journal.pntd.0007143)
Supplement: S1 Table — (DOCX) [file pntd.0007143.s001.docx]

|  | Number of variants called | variants segregating in recent Ethiopian isolates* | Average heterozygosity in parent A isolates | Average heterozygosity in parent B isolates | Average heterozygosity in putative hybrids | Heterozygosity of DM481 | Average heterozygosity of outgroups |
| --- | --- | --- | --- | --- | --- | --- | --- |
| Duplications | 169 | 95 | 0.64 | 0.60 | 0.68 | 0.69 | 0.15 |
| Deletions | 368 | 279 | 0.61 | 0.49 | 0.62 | 0.60 | 0.23 |
| Inversions | 282 | 147 | 0.63 | 0.56 | 0.69 | 0.65 | 0.17 |
| Insertions | 1 | 0 | 0 | 0 | 0 | 0 | 0 |
| Translocations | 264 | 123 | 0.44 | 0.36 | 0.42 | 0.44 | 0.16 |
