# Supplementary material for: Genomic analysis of natural intra-specific hybrids among Ethiopian isolates of Leishmania donovani
Source: PLoS Negl Trop Dis. 2020 Apr 20;14(4):e0007143. doi: 10.1371/journal.pntd.0007143 (PMC7237039; doi:10.1371/journal.pntd.0007143)
Supplement: S2 Table — Initial transition (a) and emission (b) probability matrix and trained transition (c) and emission (d) probabilities for HMM. NA represents “Not Allowed” emissions from that state. (DOCX) [file pntd.0007143.s002.docx]

(a)

| From \ To | A | B | Het |
| --- | --- | --- | --- |
| A | 0.8 | 0.1 | 0.1 |
| B | 0.1 | 0.8 | 0.1 |
| Het | 0.05 | 0.05 | 0.9 |

(b)

| State \ Symbol | A | B | Het | Non-determinate |
| --- | --- | --- | --- | --- |
| A | 0.46 | NA | NA | 0.54 |
| B | NA | 0.46 | NA | 0.54 |
| Het | NA | NA | 0.46 | 0.54 |

(c)

| From \ To | A | B | Het |
| --- | --- | --- | --- |
| A | 0.94105911 | 0.03861683 | 0.02032406 |
| B | 0.08776222 | 0.88229226 | 0.02994551 |
| Het | 0.04503840 | 0.04030465 | 0.91465696 |

(d)

| State \ Symbol | A | B | Het | Non-determinate |
| --- | --- | --- | --- | --- |
| A | 0.1592378 | NA | NA | 0.8407622 |
| B | NA | 0.1653358 | NA | 0.8346642 |
| Het | NA | NA | 0.0878155 | 0.9121845 |
